# Psychological Interventions for Reducing Interpersonal Problems and Enhancing Interpersonal Competence among Adolescents with Posttraumatic Stress Symptoms: A Meta-Analysis

**DOI:** 10.1007/s40653-026-00854-x

**Published:** 2026-03-20

**Authors:** Sabrina Ee-Ying Ong, Sayedhabibollah Ahmadi Forooshani, Zahra Izadikhah, Govind Krishnamoorthy

**Affiliations:** 1 School of Psychology and Wellbeing, University of Southern Queensland, Ipswich, Australia; 2 Adelaide University Online, Adelaide University, Adelaide, Australia

**Keywords:** Adolescents, Posttraumatic stress symptoms, Meta-analysis, Psychological interventions, Interpersonal competence, Interpersonal problems

## Abstract

This study examined the effectiveness of psychological interventions in reducing interpersonal problems and enhancing interpersonal competence among adolescents with posttraumatic stress symptoms. A systematic search of PsycInfo, PubMed, Scopus, CINAHL and Google Scholar identified 17 studies that met the inclusion criteria, with 12 effect sizes reported for interpersonal competence and 24 for interpersonal problems. Results showed similar small but statistically significant aggregated post-intervention effect for both interpersonal competence (g = 0.37, p < 0.001, 95% CI [0.20, 0.55]) and interpersonal problems (g = 0.40, p < 0.001, 95% CI [0.25, 0.54]), with intervention type and mode significantly moderating effects only for interpersonal problems. Trauma-focused Cognitive Behavioural Therapy (TF-CBT) had the largest effect on interpersonal problems, whereas skill-based interventions had the smallest. Mixed-mode interventions also showed a significantly larger effect on interpersonal problems compared to group interventions. In contrast, neither intervention type nor mode significantly moderated effects on interpersonal competence. Furthermore, insufficient follow-up data precluded evaluation of the long-term effects of interventions. This meta-analysis indicates that current interventions have small but significant aggregated post-intervention effects on both interpersonal competence and interpersonal problems, with effectiveness influenced by intervention type and mode. However, this meta-analysis is limited by small inclusion number, inclusion of heterogenous assessments, and insufficient data to examine moderating effects of gender and age. This study identified gaps in existing evidence-based interventions simultaneously targeting both interpersonal competence and interpersonal problems. Moreover, a lack of sufficient follow-up assessments raises concerns about the long-term effectiveness of existing interventions. These findings underscore the need for integrative, evidence-based interventions that target both competence and maladaptive aspects of interpersonal functioning, as well as for further high-quality studies to rigorously evaluate their long-term effectiveness across diverse adolescent populations.

## Introduction

Interpersonal functioning is vital in adolescence, as extended time spent away from home—whether at school, work, or other settings—increases both the need and likelihood for interactions with peers, romantic interests, teachers, and community members (Lam et al., 2014; Smetana et al., 2015; Xu, 2023). The quality of these social relationships influences adolescents’ social, emotional and academic development, with implications for future interpersonal outcomes (Morosan et al., 2022; Shah et al., 2024; Wilson et al., 2024). Accordingly, interpersonal functioning encompasses both positive and maladaptive dimensions. Positive interpersonal functioning, often referred to as interpersonal competence, is characterised by the ability to establish and maintain healthy relationships, engage effectively in social contexts, and navigate social challenges successfully, thereby fostering self-esteem, personal growth and adaptive behaviours (Allen et al., 2019; Bukowski et al., 2020; Ni & Chen, 2025; Pietromonaco & Collins, 2017). In contrast, interpersonal problems reflect maladaptive patterns of behaviours that undermine relationships and are associated with mental health difficulties, poorer academic performance, addiction problems, and increased risk of self-harm and suicidal behaviours (Crudgington et al., 2025; Dawood et al., 2018; Sharp & Cervantes, 2023; van den Hanenberg et al., 2025). Considered together, these constructs provide complementary perspectives on social functioning: one identifies strengths to build upon, and the other identifies difficulties that may require intervention.

Interpersonal functioning—whether in terms of competence or difficulties—is not merely the outcome of education or skill training, and it can be hindered by childhood traumatic experiences that disrupt the development of capacities essential for interpersonal functioning (Ahmadi Forooshani et al. 2021a, b). Trauma experiences, often involving threats to one’s existence, safety or integrity, can lead to a range of symptoms, including intrusive memories, avoidance of trauma-related cues, altered mood and cognition, and significant changes in arousal and reactivity, all of which may interfere with daily interpersonal functioning (American Psychological Association, 2022; Benjet et al., 2016; Herman, 2015; Knipscheer et al., 2020). Repeated or prolonged exposure to traumatic events may result in persistent difficulties with emotional regulation and negative self-identity which can consequently result in difficulties with interpersonal functioning (Kouvelis & Kangas, 2021; Niedtfeld et al., 2025; World Health Organisation, 2019), including increased interpersonal problems and reduced interpersonal competence (Elmi & Clapp, 2021; McLaughlin et al., 2020). Longitudinal findings also highlight a bidirectional relationship between trauma and interpersonal functioning, whereby reductions in PTSD symptoms predict improvements in social functioning, and improvements in interpersonal relationships are associated with decreased PTSD symptom severity (Lord et al., 2020, 2023; Wang et al., 2021). These effects are likely to be particularly pronounced during adolescence, given the central role of interpersonal functioning at this critical developmental stage.

While research on adolescents remains limited, findings from adult populations offer important insights into trauma‑related interpersonal difficulties that could extend to younger groups. Interpersonal difficulties are a central and enduring consequence of traumatic stress in adults, particularly in complex PTSD (CPTSD), where disturbances in relationships are recognised as a core feature, alongside affect dysregulation and negative self-concept (Elmi & Clapp, 2021; McLaughlin et al., 2020; World Health Organisation, 2019). Adults with PTSD and especially CPTSD, commonly experience reduced relationship satisfaction, increased interpersonal conflict, difficulties with trust and emotional intimacy, and diminished social support (Campbell & Renshaw, 2018; Scoglio et al., 2022; Wang et al., 2021). Importantly, interventions in adults demonstrate that interpersonal functioning can improve with treatment (Reich et al., 2019; Scoglio et al., 2022). Trauma-focused therapies often reduce PTSD symptoms and are accompanied by gains in social functioning (Smith et al., 2022; Swerdlow et al., 2023). Modular approaches, such as the Enhanced Skills Training in Affective and Interpersonal Regulation (Enhanced STAIR; Karatzias et al., 2023), explicitly target difficulties in interpersonal and emotional regulation and show improvements in interpersonal functioning alongside symptom reduction (Karatzias et al., 2023, 2024). While this evidence supports the changeability of interpersonal functioning, it remains unclear whether similar intervention effects can be expected in adolescent populations. Adolescence is a period of significant relational changes, during which interpersonal competence and problems may have unique trajectories and therapeutic responsiveness compared with those of adults (Cuijpers et al., 2020; Lam et al., 2014; Taubner et al., 2024).

Despite evidence from both adult and adolescent literature that posttraumatic stress symptoms adversely affect interpersonal functioning, relatively little attention has been directed towards interventions that specifically target interpersonal outcomes in adolescents with these symptoms. Existing meta-analyses on interventions for adolescents with posttraumatic stress symptoms have primarily focused on reducing PTSD and related symptoms (e.g., Gutermann et al., 2016; Lenz & Hollenbaugh, 2015; Thielemann et al., 2022) or comparing the effectiveness of different therapeutic approaches in reducing these symptoms (e.g., Hoogsteder et al., 2022; Lewey et al., 2018; Morina et al., 2016). To date, only one meta-analysis, conducted by Phillips et al. (2024), has examined the efficacy of psychological interventions on social skills and functioning amongst youth and young adult populations. Analysing 13 studies, the researchers found a small, non-significant effect favouring the best-evidenced trauma-focused interventions, TF-CBT and EMDR therapy, over control groups in improving social skills and functioning among children and youth aged five to 25. The study did not examine whether intervention characteristics, such as type, delivery mode, or session number, influenced outcomes.

Another conceptually related meta-analysis is Ahmadi Forooshani et al. (2021a, b) study, which examined the effectiveness of trauma‑focused psychological interventions on the social adjustment of refugee children and adolescents. Although the authors did not consider a PTSD diagnosis as an inclusion criterion, the samples were implicitly or explicitly treated as populations with trauma histories. The meta-analysis identified a small but significant post‑intervention effect that was not moderated by the number of sessions, age, or therapeutic approach, suggesting that the small efficacy is unlikely to improve simply by altering intervention type or dosage. The study also found no evidence of long‑term effectiveness. According to the authors (Ahmadi Forooshani et al. 2021a, b), the limited and unstable efficacy of these interventions might stem from the fact that social adjustment and interpersonal functioning were not explicitly targeted as primary therapeutic outcomes, nor were the trauma-related barriers for social adjustment systematically incorporated into intervention‑mapping processes.

Overall, evidence from both PTSD-focused and broader trauma-exposed adolescent samples indicates that interpersonal functioning is a clinically important yet under-addressed treatment outcome. Although interventions can produce modest improvements in general social functioning and adjustment, little is known about their effectiveness in reducing specific aspects, such as interpersonal problems (maladaptive patterns of behaviour that undermine relationships), or in enhancing interpersonal competence (the ability to engage effectively in social contexts). Furthermore, the narrow focus of the existing PTSD-specific meta-analysis on TF-CBT and EMDR therapy, the broad age range included, and the lack of differentiation between positive and maladaptive dimensions of social functioning highlight the need for a comprehensive evaluation of interventions that explicitly measure specific interpersonal outcomes. Further exploration of whether characteristics such as intervention type, mode and intensity moderate therapeutic outcomes in this specific context is also warranted to guide the design of more tailored interventions. These patterns underscore a critical gap in the literature that the present meta-analysis aims to address.

Recognising the importance of interpersonal competence and interpersonal problems in adolescent social functioning, and the limited attention interpersonal functioning has received in interventions, this meta-analysis evaluates the effectiveness of existing interventions for adolescents with posttraumatic stress symptoms in improving interpersonal functioning. The analysis includes all studies of interventions for adolescents experiencing posttraumatic stress symptoms that assess at least one aspect of interpersonal functioning in their outcomes. Consistent with a growing emphasis on positive, rather than solely negative, dimensions of adolescent functioning, interventions are evaluated in terms of their ability to reduce interpersonal problems and enhance interpersonal competence (Marín-Gutiérrez et al., 2024; Wang et al., 2025). Differentiating between these constructs allows identification of strengths to build upon (interpersonal competence) while addressing deficits (interpersonal problems), thereby supporting improvements in overall interpersonal functioning. Additionally, planned subgroup analyses (e.g., intervention type and delivery mode) and meta-regression analyses (e.g., number of sessions) are conducted to determine whether intervention characteristics influence interpersonal outcomes, addressing key gaps in the existing literature. Accordingly, this meta-analysis aims to determine whether existing interventions adequately address both interpersonal problems and interpersonal competence as therapeutic outcomes, and to evaluate the potential need for specialised or targeted interventions to optimise interpersonal functioning among adolescents with posttraumatic stress symptoms.

## Methods

### Literature Search

The meta-analysis was conducted in accordance with PRISMA guidelines (Liberati et al., 2009; Page et al., 2021). Multiple databases (i.e., PsycINFO, PubMed, Scopus, CINAHL) and search engines (i.e. Google Scholar) were systematically searched to identify relevant English-language articles for the current meta-analysis. The software program *Publish or Perish* was used to retrieve articles from Google Scholar (Harzing, 2007). The original search was conducted in May 2024 and updated in December 2025. A ten-year publication date limit was applied to focus on contemporary adolescent interventions, as older studies may have limited applicability to current adolescent populations due to generational changes (Al-Lawati, 2019; Grelle et al., 2023). This restriction was specified a priori in the pre-registered protocol (PROSPERO: CRD42023480411, December 2023) and adhered to ensure methodological transparency and integrity. All relevant keywords were used in the search, such as *adolescent*,* trauma*,* posttraumatic stress disorder*,* intervention*,* psychotherapy*,* interpersonal problems*,* social adjustment*, and related terms associated with the key concepts. The full search strategies for each database are provided in Appendix A. To reduce the chances of overlooking relevant studies, reference lists and citation histories of included articles were also reviewed for related peer-reviewed publications.

The inclusion criteria for the meta-analysis were as follows: (a) the studies involved psychological interventions using therapeutic and/or education-based (e.g., skills training, psychoeducation, trauma-focused interventions) methods; where intervention labels were ambiguous, studies were included only if intervention descriptions indicated the presence of core therapeutic components, (b) the participants included adolescents aged 13 to 18 years, (c) the participants had posttraumatic stress symptoms, including PTSD or CPTSD symptoms, (d) the participants did not report intellectual disabilities, (e) the participants did not have a brain or neurological disorder, (f) a measure of interpersonal functioning or an aspect of interpersonal functioning was included pre- and post-intervention, (g) sufficient statistics to compute the effect size of Hedges’ *g* were provided, and (h) original studies were published as peer-reviewed journal articles within the past 10 years. Intellectual disabilities and neurological disorders were treated as exclusion criteria only if explicitly reported in the study, where participant characteristics were not sufficiently described, no assumptions were made, and studies were retained. Studies were also excluded if they were (a) qualitative studies, (b) case studies, or (c) any other non-empirical study.

**Fig. 1:**
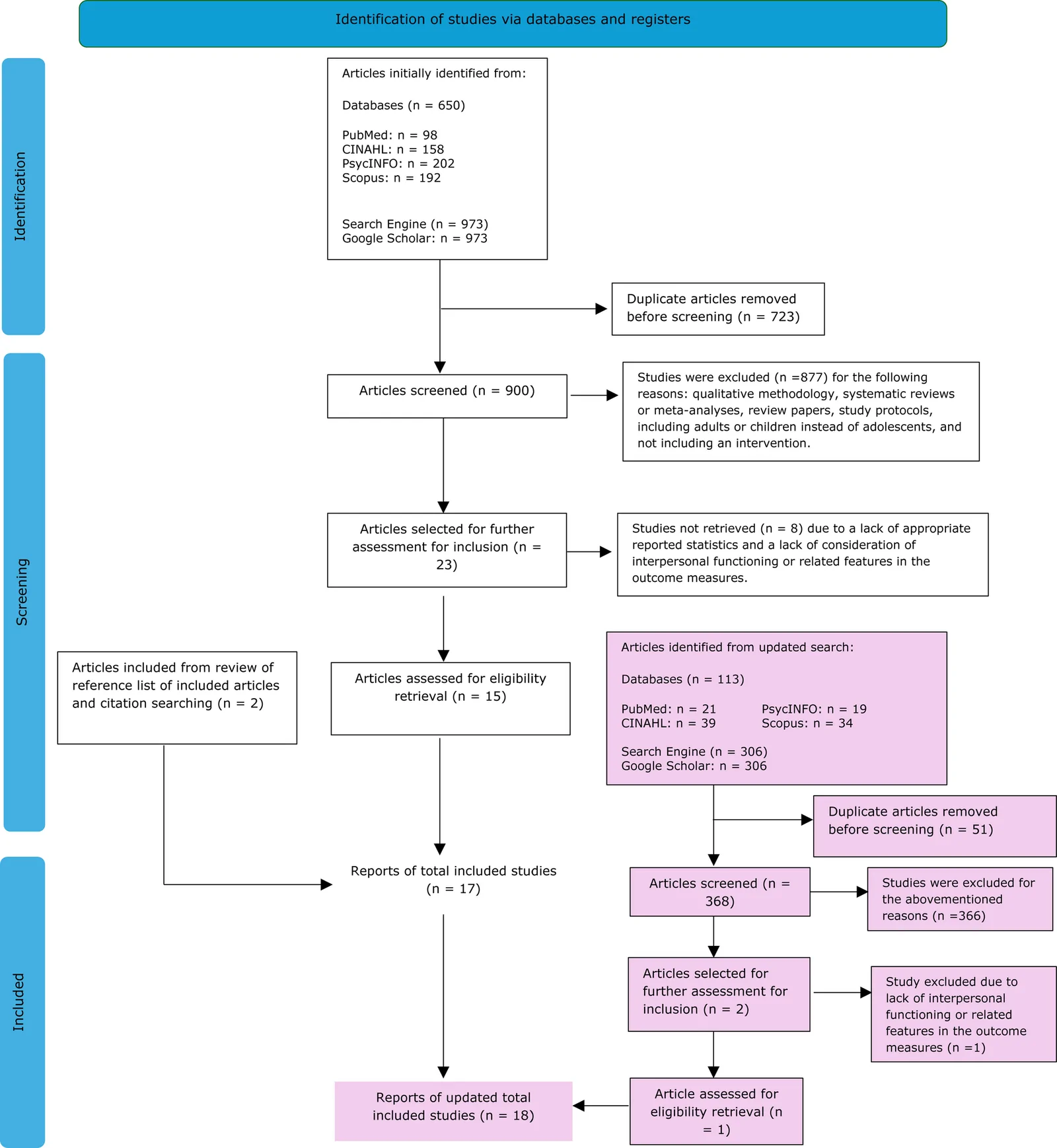
Flow diagram of the study selection process

The data extraction process is outlined in Figure 1. Database and search engine searches initially identified 1,623 articles, including 650 from databases (PubMed: 98; CINAHL: 158; PsycINFO: 202; Scopus: 192) and 973 from Google Scholar. After removing 723 duplicates using the Bond University Deduplicator tool (Forbes et al., 2024), 900 articles remained for screening against the predefined inclusion criteria using the JBI SUMMARIS software (Munn et al., 2018). The title and abstract screening resulted in the exclusion of 877 articles that did not report quantitative analyses, psychological interventions (e.g., systematic reviews, meta-analyses, or review papers), outcomes (e.g., study protocols), or adolescent samples, leaving 23 full-text articles for eligibility assessment. Of these, eight were excluded for lacking appropriate statistics to calculate effect sizes or for not including interpersonal functioning or related features in their outcome measures. An additional two studies were identified through reference list screening and citation searching, resulting in 17 included studies. An updated search identified 419 additional articles. Following the removal of 51 duplicates, 368 articles were screened, of which 366 articles were excluded at the title and abstract stage. Two full-text articles were assessed for eligibility, one of which was excluded due to the absence of an interpersonal functioning or related outcome measure. The updated search resulted in the inclusion of one additional study. Overall, 18 studies were included in the final synthesis, yielding 12 effect sizes for interpersonal competence and 24 effect sizes for interpersonal problems (*N* = 1461 individuals).

### Overview of Included Interventions

This review examined 15 specific interventions mainly aimed at reducing PTSD symptoms, with a secondary focus on improving interpersonal functioning. Most studies took place in Germany (seven) and the United States (three), while others were conducted in Spain, Australia, Canada, Chile, South Africa, Switzerland, South Korea, and India. Participants primarily came from specialised settings such as child and adolescent mental health clinics, specialised schools, and inpatient psychiatric units. Because participants often had complex profiles—having experienced at least one traumatic event and exhibiting PTSD symptoms—most studies involved more than 10 sessions of regular, intensive treatment, held daily, weekly, or twice weekly to monitor progress effectively. Some studies, however, tested shorter interventions (less than five weeks, e.g., Early Psychological Intervention for Children and Parents (EPIPAC) in Kramer & Landolt, 2014, and 3CAP in Lee & Jeon, 2021), primarily focusing on psychoeducation on trauma awareness, symptom recognition and coping strategies, rather than skill development or involving trauma-focused therapeutic processes.

The included interventions encompassed a range of psychological, therapeutic and education-based approaches, which could be broadly organised into three overlapping categories based on their primary therapeutic focus: trauma-focused processing interventions, skills-based interventions, and relational interventions.

Trauma-focused processing interventions primarily targeted the direct processing of trauma experiences and trauma-related cognitions and emotions. This category included interventions such as Developmentally Adapted Cognitive Processing Therapy (D-CPT), TF-CBT, EPIPAC, Storytelling/Story-Acting for Adolescents (STSA-A), and Mentalization-Based Treatment Group Intervention (MBT-G). Several of these approaches, including D-CPT, Cognitive Processing Therapy for Adolescents Plus (CPT-A+), and TF-CBT, explicitly emphasised the identification, challenging and modification of maladaptive trauma-related cognitions to reduce the impact of trauma on daily life. Skill-based interventions focused on structured training in coping, affect regulation and social skills to strengthen emotional and interpersonal regulation capacities. Interventions in this category included Skills Training in Affective and Interpersonal Regulation for Adolescents (STAIR-A), the Stress-Trauma-Symptoms-Arousal-Regulation-Treatment (START) program, and Yoga combined with counselling. Relational interventions focused on enhancement of safety, trust and attachment security within interpersonal contexts. Examples included in animal-assisted psychotherapy (AAP), the Rethinking Learning and Teaching Environments (ReLATE) model, interpersonal psychotherapy (IPT) and Capoeira Angola. Across several interventions, external sources of support such as therapy animals, teachers, non-offending caregivers, or mentors, were incorporated as active therapeutic elements, thereby highlighting the role of supportive relational contexts in facilitating recovery.

Although many interventions incorporated elements from more than one category, the category they are placed in reflects their primary therapeutic emphasis and provides a coherent basis for categorising interventions in subsequent analyses.

### Coding of Studies

Two independent coders coded each study included in this meta-analysis, and any discrepancies in their assessments were resolved through consensus. Searches were conducted using a broad strategy to capture studies assessing any aspect of interpersonal functioning. Following study inclusion and data extraction, all interpersonal outcome variables were reviewed collectively. During this coding process, two conceptually distinct domains of interpersonal functioning were identified based on the nature of the constructs assessed. These included interpersonal competence (e.g., prosocial behaviours, social support) and interpersonal problems (e.g. aggressive behaviours, social stress). Studies were subsequently categorised based on (a) type of intervention, (b) mode (e.g., group versus individual sessions) of intervention, (c) number of sessions, and (d) the instruments used for outcome measurement. Since only one of the included studies used a questionnaire specifically measuring interpersonal functioning (i.e., Inventory of Interpersonal Problems-SF; Horowitz et al., 2000), components of interpersonal functioning and related features were assessed using subscales from other relevant questionnaires. Consequently, a heterogeneity analysis (Borenstein et al., 2009) was conducted to assess the consistency of all assessments of interpersonal functioning.

### Measures Used by the Studies

All studies used reliable and valid measures. One-third of the studies used both the Clinician-Administered PTSD Scale for Children and Adolescents for DSM-IV (CAPS-CA; Nader et al., 1996) and the University of California Los Angeles PTSD Reaction Index for DSM-IV (UCLA-PTSD-RI; Steinberg et al., 2004) to assess posttraumatic stress symptoms. Meanwhile, one study solely employed the CAPS-CA (Kramer & Landolt, 2014), while another used only the UCLA-PTSD-RI (Gudiño et al., 2014). Other measures included the Child PTSD Symptom Scale (CPSS; Foa et al., 2001, in Goodman et al., 2023, Gudiño et al., 2016 and Guerra et al., 2024), Posttraumatic Stress Disorder Checklist, Parent Report (PCL-PR; Weather & Ford, 1996, in Diggins, 2021), Child and Adolescent Trauma Screening (CATS; Sachser et al. 2017a, b, in Dixius & Möhler, 2021), and the Children’s Impact of Trauma Events Scale II (CITES II; Wolfe, 2002) PTSD subscale (in Hébert & Amédée, 2020).

Regarding measurements related to interpersonal functioning, the only full-scale measure – Inventory of Interpersonal Problems, short form (IIP-SF; Horowitz et al., 1988; Soldz et al., 1995) – was used. Subscales of the Behaviour Assessment System for Children (BASC; Reynolds & Kamphaus, 1992, in Balluerka et al., 2015, and Gudiño et al., 2016), the Strengths and Difficulties Questionnaire (SDQ; Goodman, 2001; in Diggins, 2021, and Mom et al., 2019), and the FEEL-KJ (Goldschmidt et al., 2006, in Dixius & Möhler, 2021) assessed both interpersonal competence and problems. Subscales of the Adolescent Attachment Questionnaire (AAQ; Westen et al., 2006, in Goodman et al., 2023) and KidCOPE (Spirito et al., 1988, in Gudiño et al., 2014) measured only interpersonal competence. In contrast, subscales of the Child and Behaviour Checklist (CBCL; Achenbach & Rescorla, 2001, in Goldbeck et al., 2016, Hébert & Amédée, 2020, and Kramer & Landolt, 2014), the Youth Self-Report (YSR; Achenbach, 1991, in Lee & Jeon, 2021, Rosner et al., 2019, and Vogel et al., 2020), Experiences in Close Relationships - Revised Questionnaire (ECR-R; Ehrenthal et al., 2009, in Rimane et al., 2021), and the Emotional Maturity Scale (Singh & Bhargava, 1990, in Mukherjee, 2019) were used to evaluate interpersonal problems.

### Methodological Quality Assessment

The Effective Public Health Practice Project Quality Assessment Tool for Quantitative Studies (EPHPP; McMaster University, 2008) was used for quality assessment in this study. The EPHPP evaluates the quality of quantitative studies to support the synthesis of knowledge from these studies (Tanner, 2019). Two authors of this research independently evaluated each study included in the meta-analysis using the six domains of the EPHPP: selection bias, study design, control of confounders, blinding, data collection methods, and participant withdrawals and dropouts (McMaster University, 2008). Based on the scores for each domain, the quality of each study was categorised as strong (none of the elements rated as weak), moderate (one element rated as weak), or weak (two or more elements rated as weak). The findings are presented in Table 1 of the Appendix. The blinding domain posed challenges for most studies, affecting their quality ratings. Despite this, most studies were regarded as moderate in quality, with one rated as weak and three as strong. It is important to note that this scale is used in this study to provide an overview of the quality of studies included in the meta-analysis. It is neither an inclusion criterion for the meta-analysis nor regarded as a necessary exclusion or inclusion criterion for meta-analytical assessments in general (Borenstein et al., 2009).

**Table 1:** Effective public health practice project (EPHPP) Quality assessment tool for quantitative studies (*n* = 17)

	Component Ratings						Global Rating
	(A) Selection Bias	(B) Study Design	(C) Confounders	(D) Blinding	(E) Data Collection Method	(F) Participant withdrawals and dropouts	
Balluerka et al., 2015	2 Moderate	1 Strong	1 Strong	3 Weak	1 Strong	1 Strong	2 Moderate
Rosner et al., 2019	2 Moderate	1 Strong	1 Strong	2 Moderate	1 Strong	2 Moderate	1 Strong
Fischer at al., 2022	2 Moderate	2 Moderate	1 Strong	2 Moderate	1 Strong	2 Moderate	1 Strong
Rimane et al., 2021	2 Moderate	1 Strong	1 Strong	3 Weak	1 Strong	2 Moderate	2 Moderate
Diggins, 2021	2 Moderate	2 Moderate	1 Strong	3 Weak	1 Strong	2 Moderate	2 Moderate
Dixius & Möhler, 2021	2 Moderate	2 Moderate	1 Strong	3 Weak	1 Strong	2 Moderate	2 Moderate
Goodman et al., 2023	2 Moderate	1 Strong	1 Strong	3 Weak	1 Strong	2 Moderate	2 Moderate
Gudiño et al., 2014	2 Moderate	2 Moderate	1 Strong	3 Weak	1 Strong	1 Strong	2 Moderate
Gudiño et al., 2016	2 Moderate	1 Strong	1 Strong	3 Weak	1 Strong	2 Moderate	2 Moderate
Hébert & Amédée, 2020	2 Moderate	2 Moderate	1 Strong	3 Weak	1 Strong	1 Strong	2 Moderate
Kramer & Landolt, 2014	2 Moderate	1 Strong	1 Strong	3 Weak	1 Strong	1 Strong	2 Moderate
Lee & Jeon, 2021	2 Moderate	1 Strong	1 Strong	3 Weak	1 Strong	2 Moderate	2 Moderate
Mom et al., 2019	2 Moderate	2 Moderate	1 Strong	3 Weak	1 Strong	2 Moderate	2 Moderate
Mukherjee, 2019	2 Moderate	2 Moderate	1 Strong	3 Weak	2 Moderate	2 Moderate	2 Moderate
Sachser et al. 2017a, b	2 Moderate	2 Moderate	1 Strong	3 Weak	1 Strong	1 Strong	2 Moderate
Goldbeck et al., 2016	2 Moderate	1 Strong	1 Strong	2 Moderate	1 Strong	1 Strong	1 Strong
Vogel & Rosner, 2020	3 Weak	2 Moderate	1 Strong	3 Weak	1 Strong	2 Moderate	3 Weak

### Statistical Analysis

In this study, the meta-analyses were conducted using Hedges’ *g* to compute aggregated effect sizes. Hedges’ *g* can be calculated from mean differences and pooled standard deviations or derived from other reported statistics (e.g., *t* or *F* values). Mean differences in effect sizes through a random-effects model were used to compute effect sizes and potential moderators. Random (or mixed) effects analysis is recommended when results from multiple random samples should be generalised to a broader population (Hedges & Vevea, 1998; Konstantopoulos, 2006).

The type and mode of intervention, as well as the number of sessions, were examined as potential moderators. Meta-regression was used to assess the only continuous moderator: the number of sessions. For the type of intervention, there were not enough studies for each specific type of intervention (i.e., 12 interventions with one study each) to conduct meaningful subgroup analyses. Therefore, interventions were grouped into broader, conceptually defined categories to increase statistical power.

Trauma-focused Cognitive Behaviour Therapy (**TF-CBT**) was treated as a distinct category when studies explicitly employed the manualised TF-CBT protocol, given its established evidence base and frequent examination in prior meta-analyses within this population. Cognitive Behaviour Therapy (**CBT**) was classified separately when it did not include direct trauma processing, allowing differentiation from TF-CBT. Interventions that primarily utilised bodily movement as the central therapeutic mechanism were categorised as **Movement Interventions**. All remaining interventions were classified using the three categories identified in the intervention overview, based on their dominant therapeutic focus: **Processing-based**, **Skills-Based**, and **Relational** Interventions. Processing-based interventions included approaches other than TF-CBT that directly engaged in the processing of trauma experiences and trauma-related cognitions and emotions. Skills-based interventions are primarily aimed at strengthening emotional and interpersonal regulation capacities through structured skill development and practice. Relational interventions emphasised enhancing relational safety, attachment security, and interpersonal functioning. These groupings were based on the primary therapeutic emphasis of each intervention rather than on intervention labels alone.

The mode of intervention was classified according to the primary format of delivery: **Individual** (one-to-one sessions), **Group** (sessions delivered in a group format), or **Mixed** (a combination of individual and conjoint or group sessions). Subgroup analyses were conducted for the categorical moderators of intervention type and intervention mode.

All analyses were conducted using the Comprehensive Meta-Analysis (CMA; Borenstein, 2022), Version 4. The heterogeneity was assessed using Q statistics, while publication bias was examined using the Classic Fail-safe N, which estimates the number of unpublished null-effect studies required to reduce the computed aggregated effect size to a non-significant value (*p* < 0.05; Rosenthal, 1979, 1991).

## Results

Most studies included in the meta-analysis used validated measures to assess PTSD symptoms and featured multiple subscales related to interpersonal functioning (see Methods for details). Each subscale was considered a separate effect size. Subscales that assessed positive aspects of interpersonal functioning (e.g., prosocial behaviours and social support subscales) and reflected the ability to interact effectively with others (Gurtmann, 1999; Spitzberg & Cupach, 2012) were categorised as *interpersonal competence*. Conversely, subscales indicating difficulties in interpersonal functioning (e.g., aggressive behaviours and social stress (Erozkan, 2013; Horowitz et al., 1988, 1993) were classified as *interpersonal problems*. The specific subscales assigned to each category are provided in Tables 2 and 3, respectively.

### Interpersonal Competence

According to the heterogeneity test, the effect sizes used as the outcome for interpersonal competence were not significantly heterogeneous, allowing them to be regarded as a single holistic outcome variable (*Q* = 0.49, *p* > 0.05). Table 2 presents studies related to the constructs of interpersonal competence.

**Table 2:** Studies with Interpersonal Competence constructs

Study	Study Design	Sample	Age Range	Country	Intervention	Type of Intervention	Duration	Number of sessions	Instrument	Interpersonal construct
Balluerka et al., 2015	Quasi-experimental design	Treatment = 39 Control = 24	12–17 years	Spain	Animal-assisted psychotherapy (AAP)	Mixed	12 weeks	Group = 23 sessions Individual = 11	Behaviour Assessment System for Children (BASC) subscales	Personal adjustment (SRP)
										Adaptive Skills (PRS)
										Adaptive Skills (TRS)
Diggins, 2021	Quasi-experimental design	Treatment 1 = 8 Treatment 2 = 10	9–16 years	Australia	Rethinking Learning and Teaching Environments (ReLATE) intervention model	Individual	12 months	Not specified	Strengths & Difficulties Questionnaire (SDQ) subscale	Prosocial behaviours
Dixius & Möhler, 2021	Quasi-experimental design	Treatment = 66	13–18 years	Germany	Stress-Trauma-Symptoms-Regulation-Treatment (START) program	Group	5 weeks	10 sessions	FEEL-KJ: Questionnaire for the Assessment of Emotional Regulation in Children and Adolescents subscale	Social support
Goodman et al., 2023	Quasi-experimental design	MBT-G = 58 STSA-A = 51	13–14 years	United States of America	Storytelling/Story-Acting for Adolescents (STSA-A) Mentalisation-Based Treatment Group Intervention (MBT-G)	Group	8 months	32 sessions	Adolescent Attachment Questionnaire (AAQ)	Attachment security
Gudiño et al., 2014	Quasi-experimental design	Treatment = 38	12–17 years	United States of America	Brief Skills training in affective and interpersonal regulation (STAIR)-A	Group	Not specified	3–36 sessions	KID-COPE	Coping skills used for interpersonal stressors
										Coping efficacy with interpersonal stressors
Gudiño et al., 2016	Quasi-experimental design	Treatment = 23 Control = 23	11–16 years	United States of America	Skills training in Affective and Interpersonal Regulation for adolescents (STAIR-A)	Group	16 weeks	16 sessions	Behaviour Assessment System for Children (BASC) subscales	Interpersonal Relations
Mom et al., 2019	Quasi-experimental design	Treatment = 32	12–18 years	Australia	Capoeira Angola	Group	9 months	30 sessions	Strengths & Difficulties Questionnaire (SDQ) subscales	Prosocial behaviours

**Fig. 2:**
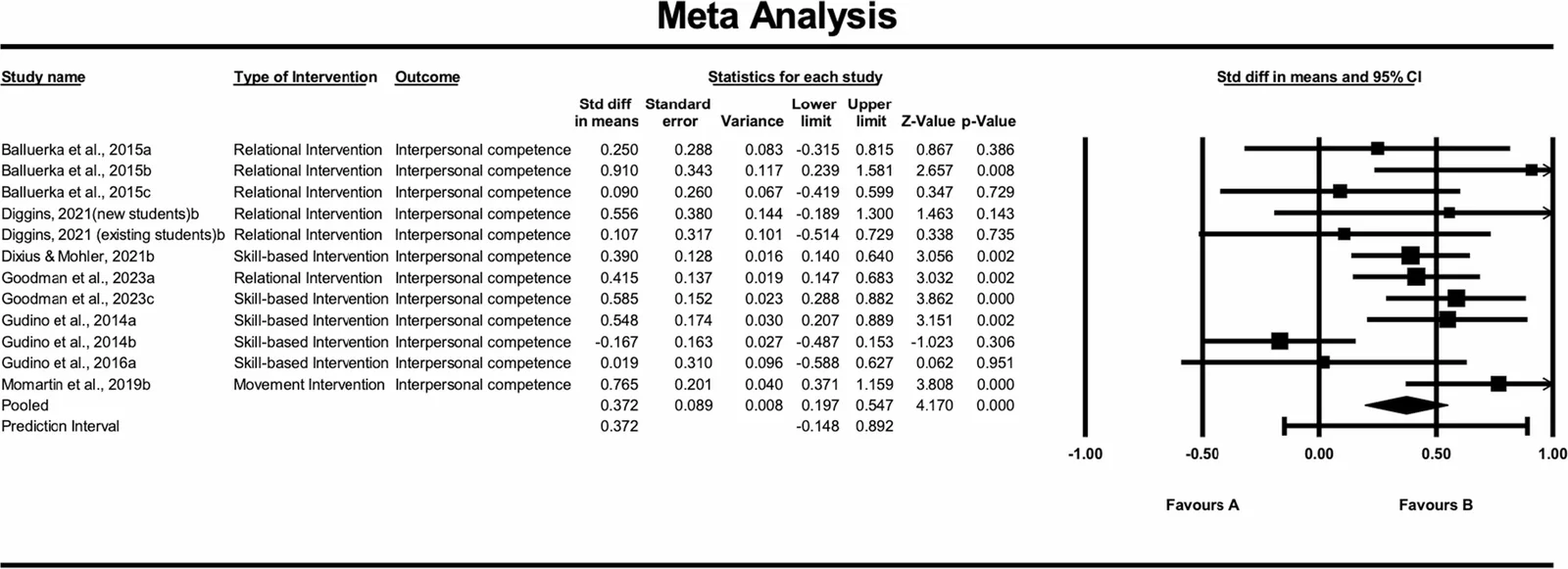
Forest plot for interpersonal competence studies

The post-intervention aggregated effect size for studies examining interpersonal competence was computed as g = 0.37, *p* < 0.001, 95% CI [0.20, 0.55] (see Fig. 2 for a forest plot showing all studies investigating interpersonal competence). According to Cohen’s guidelines, the effect size is considered small (Cohen, 1992).

**Fig. 3:**
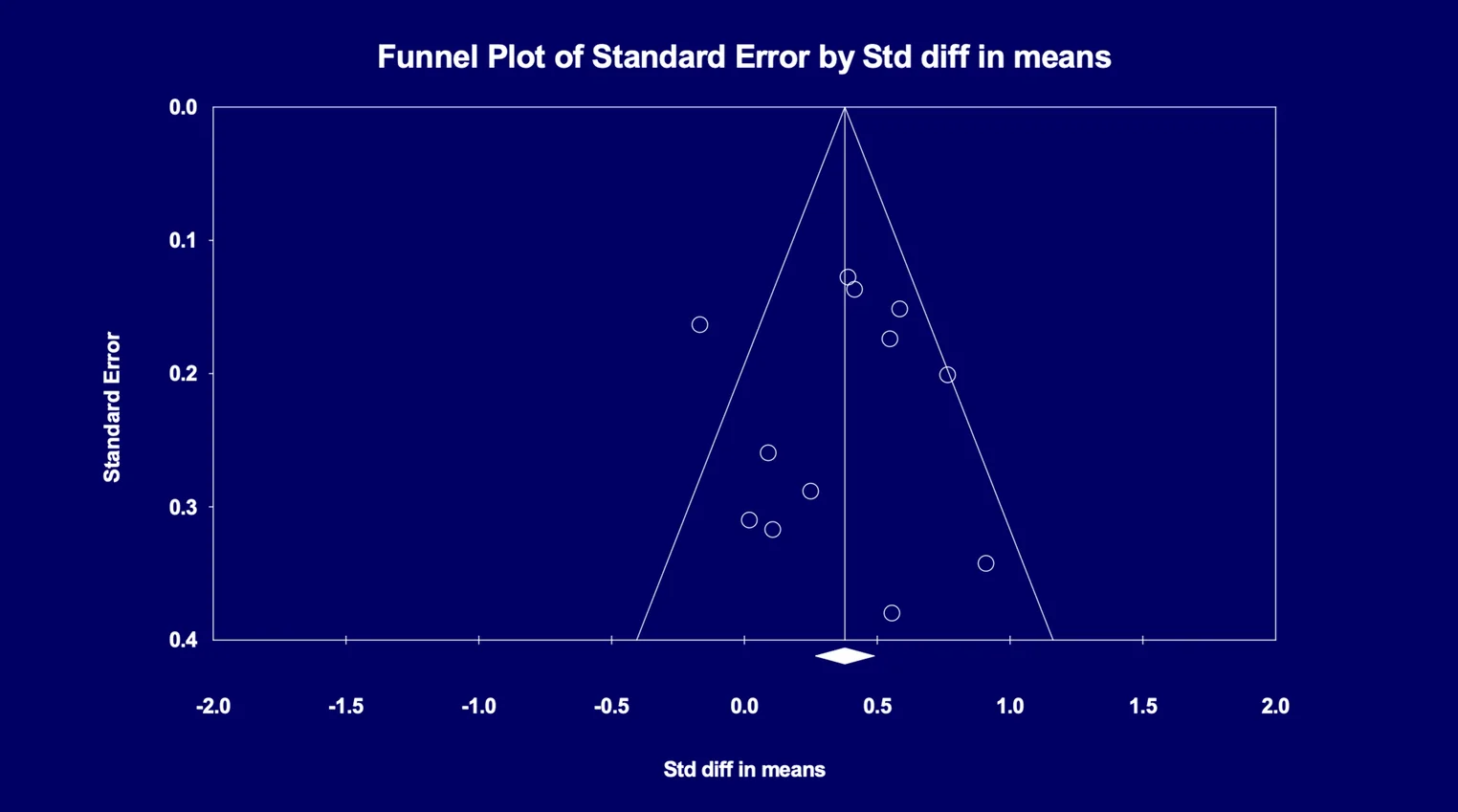
Funnel plot for interpersonal competence studies

Based on classic fail-safe *N* analysis (Rosenthal, 1979, 1991) for studies examining interpersonal competence, 110 studies with non-significant effect sizes are needed to reduce this effect size to a non-significant value (*p* > 0.05). Visual inspection of the funnel plot (Fig. 3) did not suggest substantial asymmetry, indicating a low likelihood of publication bias. Therefore, these results indicate a small but statistically significant aggregated efficacy of the included interventions in enhancing the interpersonal competence of adolescents with posttraumatic stress symptoms.

The results of the meta-regression analysis showed no significant moderating effect of the number of therapeutic sessions on studies investigating interpersonal competence (*b* = 0.0071, SE = 0.015, *p* = 0.62). This indicates that increasing the number of sessions may not lead to greater improvements in interpersonal competence. Subgroup analyses were conducted using categorical moderators of intervention type and mode of intervention. Three intervention types—Relational, Movement, and Skills-based—were identified in studies assessing interpersonal competence outcomes. The moderating effect of intervention type on improving interpersonal competence was not significant (Q = 3.84, *p* = 0.15), indicating that no intervention type demonstrated a statistically superior effect on interpersonal competence. Only one study in this analysis employed a movement-based intervention; therefore, results for this category are descriptive only and do not permit meaningful subgroup comparison. To examine whether inclusion of this single study influenced the overall moderator test, a sensitivity analysis was conducted excluding the movement-based intervention. The moderating effect of intervention type remained non-significant (Q = 0.16, *p* = 0.69), indicating that the overall conclusion was robust to excluding this study.

Similarly, the moderating effect of the intervention mode on enhancing interpersonal competence was not significant (Q = 0.11, *p* = 0.94), suggesting that the interventions can be equally effective whether delivered through individual sessions, group sessions, or a combination of both individual and group or conjoint sessions. These findings indicate that improvements in interpersonal competence may be achieved across a range of intervention approaches and delivery formats, although these results should be interpreted cautiously given the limited number of studies within each subgroup.

### Interpersonal Problems

Based on the heterogeneity test, the effect sizes used as the outcome variable for interpersonal problems were not significantly heterogeneous and could therefore be regarded as one holistic outcome variable (*Q* = 2.87, *p* > 0.05). Table 3 displays the studies with constructs related to interpersonal problems.

**Table 3:** Studies with Interpersonal Problems constructs

Study	Study Design	Sample	Age Range	Country	Intervention	Types of Intervention	Duration	Number of sessions	Instrument	Interpersonal constructs
Balluerka et al., 2015	Quasi-experimental design	Treatment = 39 Control = 24	12–17 years	Spain	Animal-assisted psychotherapy (AAP)	Mixed	12 weeks	Group sessions = 23 Individual = 11 sessions	Behaviour Assessment System for Children (BASC) subscales	School Maladjustment (SRP)
Rosner et al., 2019	Randomised controlled trial	Treatment = 44 Control = 44	14–21 years	Germany	Developmentally adapted cognitive processing therapy (D-CPT)	Individual	16 to 20 weeks	36 sessions	Youth Self-report (YSR) subscale	Behaviour problems
Fischer et al., 2022	Quasi-experimental design	Treatment = 44	17.5–18.8 years	Germany	Developmentally adapted Cognitive Processing Therapy (D-CPT)	Individual	Unknown	36 sessions	Diary cards for urge to engage in behaviours (0 to 5 rating) and actual actions (yes/no)	Aggressive behaviour - urge to engage in behaviours
										Aggressive behaviour – actual action
Rimane et al., 2021	Randomised controlled trial	Treatment = 43 WL/TA = 42	14–21 years	Germany	Developmentally adapted cognitive processing therapy (D-CPT)	Individual	16 to 20 weeks	30 (50-min) sessions + 6 optional joint sessions with caregiver/crisis intervention	Experiences in Close Relationships -Revised Questionnaire (ECR-R)	Attachment-related (AR) anxiety
										Attachment- related (AR) avoidance
Diggins, 2021	Randomised controlled trial	Sample 1 (new) = 8 Sample 2 (existing) = 10	9–16 years	Australia	Rethinking Learning and Teaching Environments (ReLATE) intervention model	Individual	12 months	Not specified	Strengths & Difficulties Questionnaire (SDQ) subscale	Peer problems
Dixius & Möhler, 2021	Quasi-experimental design	Treatment = 66	13–18 years	Germany	Stress-Trauma-Symptoms-Regulation-Treatment (START) program	Group	5 weeks	10 (60-min) sessions	FEEL-KJ: Questionnaire for the Assessment of Emotional Regulation in Children and Adolescents	Aggressive behaviour
									subscale	
Gudiño et al., 2016	Quasi-experimental design	Treatment = 23 Control = 23	11–16 years	United States of America	Skills training in Affective and Interpersonal Regulation for adolescents (STAIR-A)	Group	16 weeks	16 sessions	Behaviour Assessment System for Children (BASC) subscales	Social Stress
Hébert & Amédée, 2020	Quasi-experimental design	CPTSD = 79 PTSD = 170 Resilient = 77	6–14 years	Canada	Trauma-focused cognitive-behavioural therapy (TF-CBT)	Mixed	Unknown	14–19 sessions	Child Behaviour Checklist (CBCL) subscale	Interpersonal problems
										Externalising behaviours
Kramer & Landolt, 2014	Randomised Controlled Trial	Treatment = 54 Control = 54	7–16 years	Switzerland	Early psychological intervention for children and parents (EPICAP)	Individual	2 weeks	2 sessions	Child Behaviour Checklist (CBCL) subscale	Externalising behaviours
Lee & Jeon, 2021	Quasi-experimental design	Treatment = 14 Control = 14	12–20 years	South Korea	3CAP Program	Group	4 weeks	8 sessions (2 sessions per week)	Youth Self-report (YSR) subscale	Aggression
Mom et al., 2019	Quasi-experimental design	Treatment = 32	12–18 years	Australia	Capoeira Angola	Group	9 months	30 sessions	Strengths & Difficulties Questionnaire (SDQ) subscales	Peer problems
Mukherjee, 2019	Quasi-experimental design	Treatment group = 140	Unspecified	India	Yoga with counselling	Mixed	Not specified	Not specified	Emotional Maturity Scale	Social Maladjustment
Sachser et al. 2017a, b	Quasi-experimental design	Treatment = 27	7–17 years	Germany	Trauma-Focused CBT (TF-CBT)	Mixed	4 months	12 weekly 90-minute sessions	Clinician-Administered PTSD Scale for Children and Adolescents for DSM-IV (CAPS-CA) University of California Los Angeles PTSD Reaction Index for DSM-IV (UCLA-PTSD-RI)	Interpersonal problems
Goldbeck et al., 2016	Randomised Controlled Trial	Treatment = 76 Control = 83	7–17 years	Germany	Trauma-Focused CBT (TF-CBT)	Mixed	4 months	12 weekly 90-minute sessions	Child Behaviour Checklist (CBCL) subscale	Externalising behaviours
Vogel & Rosner, 2020	Quasi-experimental design	Treatment group = 17	14–21 years	Germany	CPT + A (minor changes to CPT)	Mixed	Mean duration = 26.24	15 50-min sessions	Youth Self-report (YSR) subscale	Externalising behaviours
								(Up to 3 further optional sessions)		
Guerra et al., 2024	Quasi-experimental design	TF-CBT Treatment group = 23 IPT Treatment group = 22 Control = 22	13–17 years	Chile	Trauma-Focused CBT (TF-CBT) Interpersonal Psychotherapy	Group	12 weeks	12 1.5–2 h sessions	Inventory of Interpersonal Problems, Short Form (IIP-SF)	Interpersonal problems

**Fig. 4:**
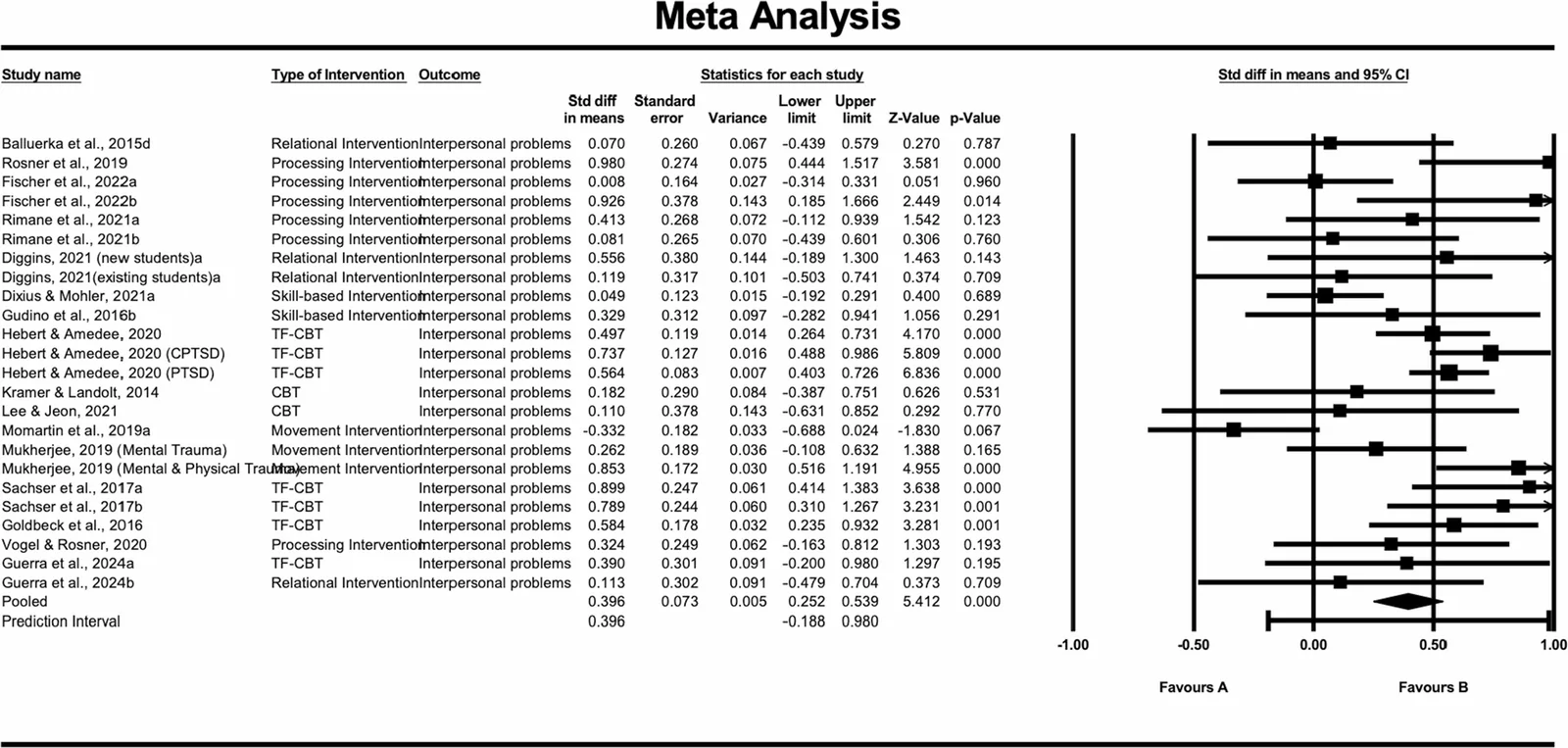
Forest plot for interpersonal problems studies

The post-intervention aggregated effect size for studies investigating interpersonal problems was computed as *g* = 0.40, *p* < 0.001, 95% CI [0.25, 0.54] (see Fig. 4 for a forest plot including all studies investigating interpersonal problems), indicating a small effect size according to Cohen’s guidelines (Cohen, 1992).

**Fig. 5:**
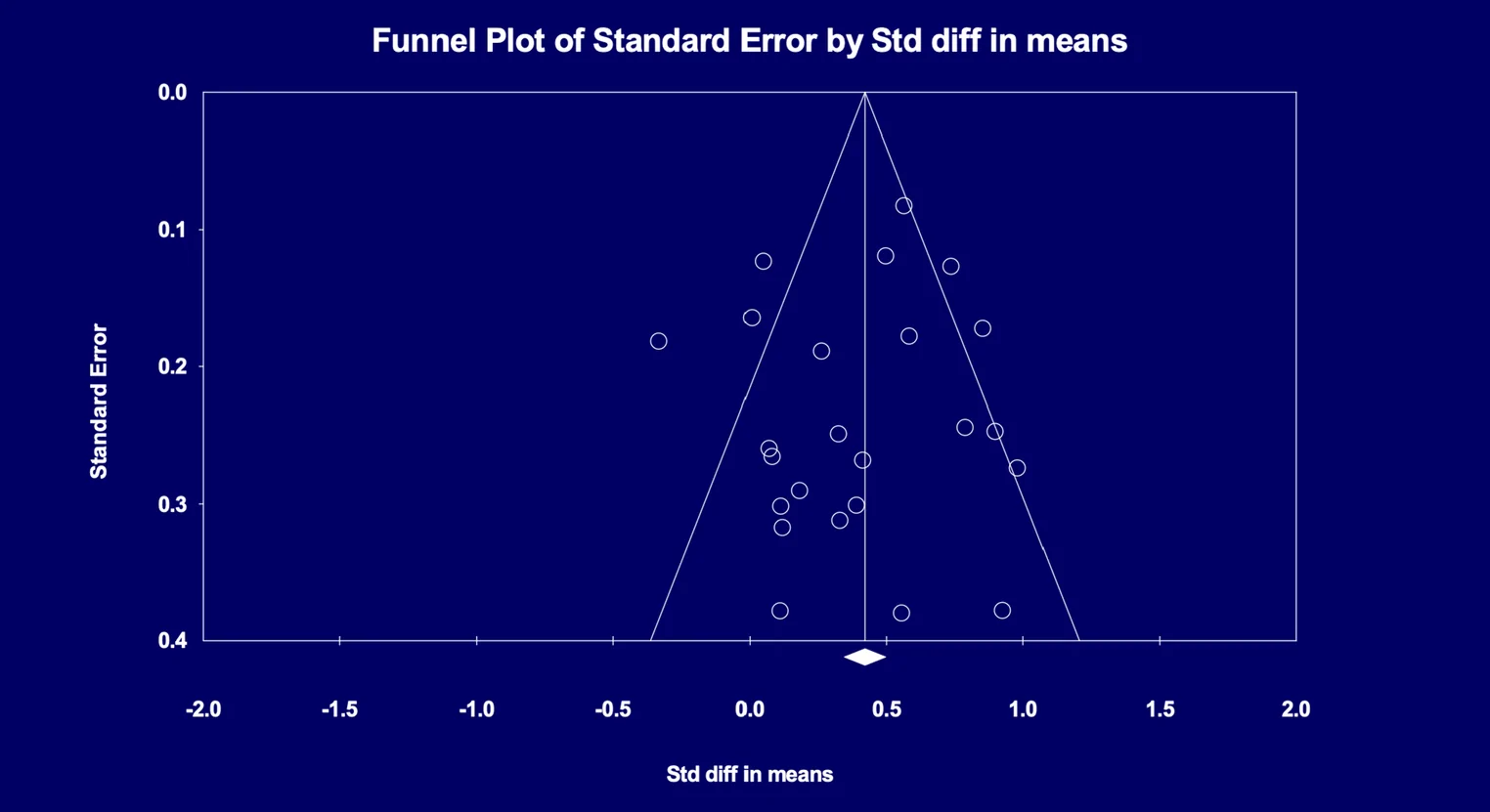
Funnel plot for interpersonal problems studies

Based on the classic fail-safe *N* analysis (Rosenthal, 1979, 1991) for studies examining interpersonal problems, 548 studies with non-significant effect sizes are required to reduce this effect size to a non-significant value (*p* > 0.05). Visual inspection of the funnel plot (Fig. 5) did not suggest substantial asymmetry, indicating a low likelihood of publication bias. Therefore, the findings suggest a small but statistically significant general efficacy of the included interventions in reducing interpersonal problems among adolescents with posttraumatic symptoms.

The results of the meta-regression analysis showed no significant moderating effect of the number of therapeutic sessions on studies examining interpersonal problems (*b* = -0.0053, SE = 0.0073, *p* = 0.47). The subgroup analyses indicated a significant moderating effect of the type (Q = 23.67, *p* < 0.001) and mode (Q = 19.10, *p* < 0.001) of intervention in reducing interpersonal problems.

**Fig. 6:**
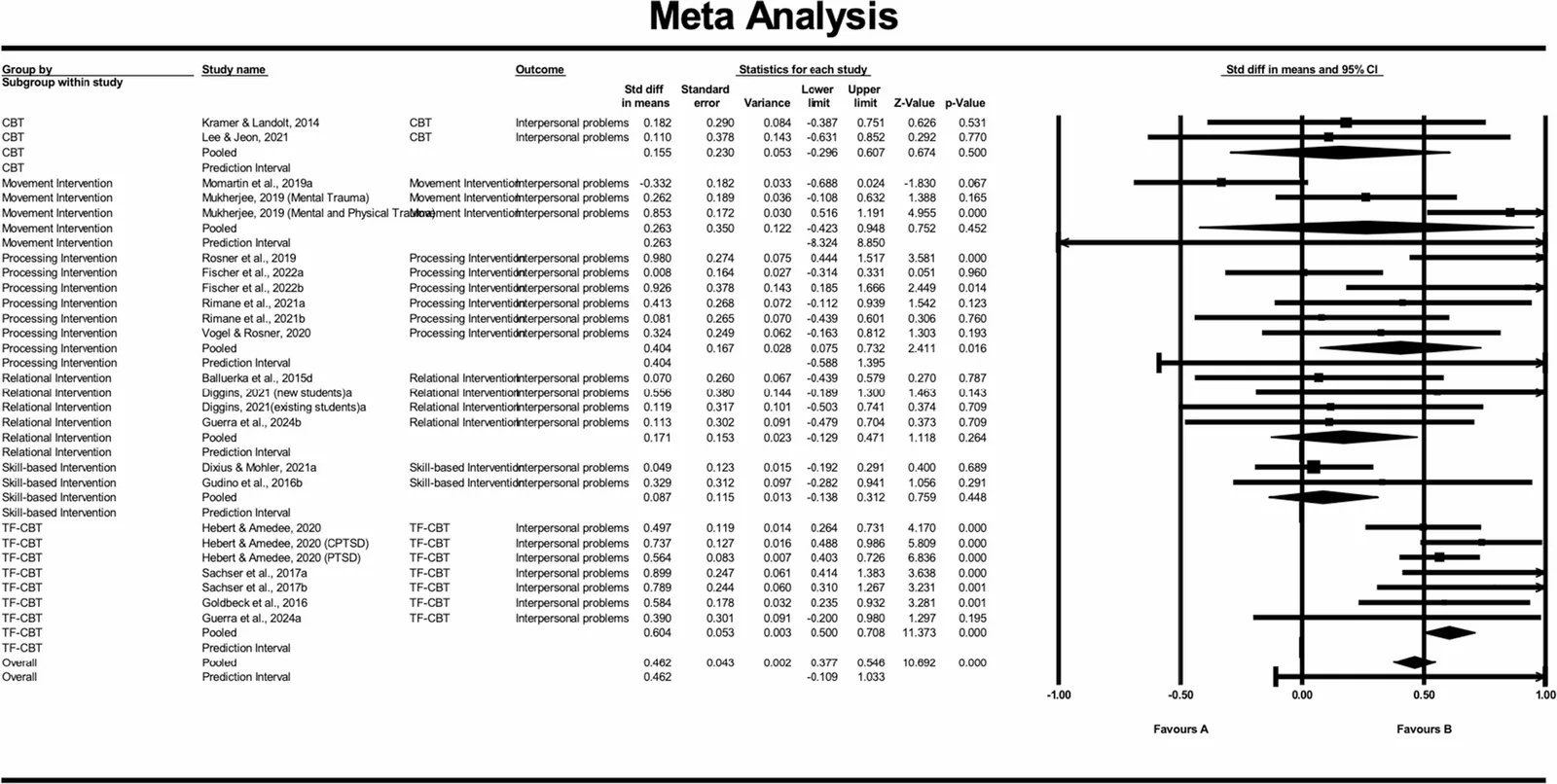
Forest plot for type of intervention subgroup analysis of interpersonal problems studies

Accordingly, the largest aggregated effect size belonged to TF-CBT (g = 0.604, *p* < 0.001) for the type of intervention (see Fig. 6 for a forest plot for type of intervention subgroup analysis of interpersonal problems studies), and with a mixed mode of delivery (g = 0.590, *p* < 0.001) for the mode of intervention. The only significant follow-up pairwise subgroup analysis found that TF-CBT was significantly more effective than skill-based interventions, which showed the smallest aggregated effect size among the interventions. Regarding the mode of intervention, mixed mode delivery did not differ significantly from individual delivery, but both showed a significantly larger effect compared to group delivery.

### Studies with Both Aspects of Interpersonal Functioning

This meta-analysis examined two aspects of interpersonal functioning: interpersonal competence and interpersonal problems. Therefore, interest lies in the aggregated effect size from studies that included both aspects. Only five studies (Balluerka et al., 2015; Diggins, 2021, Dixius & Mohler, 2021, Gudino et al., 2016, and Mom et al., 2019) examined both aspects, demonstrating a small effect size for the aggregated outcome of interpersonal functioning, which regards interpersonal competence and interpersonal problems as a single variable (g = 0.24, *p* = 0.009, 95% CI [ 0.06, 0.43]). These studies included relational, skill-based and movement interventions. This underscores the need for further research to identify intervention approaches that more effectively target both interpersonal competence and interpersonal problems, including the potential value of trauma-focused methods.

## Discussion

This meta-analysis examined the effectiveness of psychological interventions on interpersonal functioning in adolescents with posttraumatic stress symptoms, distinguishing between two dimensions of interpersonal functioning: interpersonal competence and interpersonal problems. The results indicated a small but statistically significant post-intervention effect for both interpersonal competence and interpersonal problems. While the type and mode of intervention significantly moderated the outcome for interpersonal problems, there was no moderator that meaningfully influenced interpersonal competence. Also, due to limited follow-up data, the long-term effects of these interventions could not be assessed.

These findings align with recent research suggesting that interpersonal difficulties in trauma-exposed adolescents often persist even after trauma symptom reduction and may represent a distinct therapeutic target (e.g., Swerdlow et al., 2023). Trauma-focused interventions are well-established in reducing PTSD, yet their impact on broader social and interpersonal functioning appears more modest and inconsistent (Phillips et al., 2024; Swerdlow et al., 2023). The small but significant effects observed in the present meta-analysis suggest that when interventions primarily target trauma symptoms, improvements in interpersonal functioning may occur only indirectly and to a limited extent, reinforcing the need for interventions that explicitly target interpersonal processes directly.

Comparable patterns are evident in meta-analytic evidence on the effectiveness of psychological interventions for social adjustment among trauma-exposed child and adolescent refugees (Ahmadi Forooshani et al. 2021a, b). Although the study did not explicitly assess PTSD symptoms, it similarly reported small, significant improvements in social adjustment following interventions, which were not moderated by participant age, number of sessions, or therapeutic approaches, echoing the limited influence of these factors observed in the present meta-analysis. The effects observed in the refugee study were smaller than those in the current analysis, likely reflecting methodological differences, such as the exclusive inclusion of controlled studies, and the unique challenges faced by refugee populations, including complex trauma histories and barriers to social functioning.

Despite the small intervention effects observed, these findings parallel patterns observed in adult PTSD and CPTSD research, which suggest that interpersonal functioning of young people with trauma histories can be improved with tailored psychological interventions but often remains only partially addressed (Reich et al., 2019; Scoglio et al., 2022). In adults, interpersonal problems persist following trauma-focused treatment, particularly among those with histories of chronic or interpersonal trauma (Cloitre et al., 2013; Swerdlow et al., 2023). Accordingly, adult CPTSD treatment models emphasised that trauma-related interpersonal difficulties require targeted intervention components, such as emotion regulation, relational skills training and phase treatment approaches (Ahmadi Forooshani et al., 2022; Cloitre et al., 2013; Karatzia et al., 2024; Izadikhah et al., 2025). The present findings similarly indicate that interpersonal problems may persist following trauma-focused treatment in adolescents when the interventions are not tailored to specifically target this therapeutic outcome. This underscores the potential value of adapting evidence-based tailored adult intervention components for younger populations in a developmentally sensitive manner.

The current meta-analysis builds on Phillips et al. (2024), the only other research specifically examining the effects of interventions on social functioning in adolescents with posttraumatic stress symptoms. Similar to Phillips et al. (2024), most studies relied on subscales from various measures to assess interpersonal functioning. However, the findings differ: while Phillips et al. (2024) reported a small, non-significant effect when focusing solely on TF-CBT and EMDR therapy, the present study identified small but statistically significant aggregated post-intervention effects on both aspects of interpersonal functioning—interpersonal competence and interpersonal problems. Several factors may explain this discrepancy. First, the present study distinguished between interpersonal competence and interpersonal problems, rather than treating interpersonal functioning as a unitary construct, underscoring the importance of conceptual clarity. Second, the narrow age range (13 to 18 years) may have reduced developmental heterogeneity. Third, the inclusion of diverse intervention types beyond TF-CBT and EMDR therapy and study designs not being limited to RCTs may have captured meaningful changes in interpersonal functioning not detected in more restricted designs. These findings addresses Phillips et al. (2024)’s question about whether more diverse interventions could lead to different outcomes, suggesting that, beyond the most supported PTSD treatments, a variety of interventions may be necessary to improve interpersonal functioning in adolescents with trauma symptoms.

Examining interpersonal functioning through interpersonal competence and interpersonal problems aligns with the contemporary shift towards focusing on positive adolescent functioning rather than just negative aspects (Marín-Gutiérrez et al., 2024; Wang et al., 2025). Since both aspects show small but statistically significant aggregated post-intervention effects, there is interest in how interventions may differ in their effectiveness for each. The results showed that the type and mode of interventions influenced their effectiveness in different ways. Both the type and mode of interventions significantly moderated reductions in interpersonal problems, while neither enhanced interpersonal competence, indicating that the mechanisms through which change occurred in these two aspects differed.

Intervention type and mode significantly moderated outcomes for interpersonal problems but not interpersonal competence. TF-CBT demonstrated a significant medium effect on interpersonal problems, with an aggregated effect size greater than other intervention types. This finding is consistent with theoretical models suggesting that trauma-related interpersonal difficulties are often relationally maintained through maladaptive trauma-related beliefs and behaviours, which TF-CBT directly addresses via trauma processing and cognitive restructuring (APA, 2017; Foa et al., 2000). By targeting these maintaining mechanisms, TF-CBT contributed to its greater effectiveness, as reflected in the medium effect size observed in this study. Notably, the meta-analysis of child and adolescent refugees (Ahmadi Forooshani et al. 2021a, b a) found that interventions incorporating trauma-processing components were more effective than other approaches, reinforcing the importance of directly addressing trauma-related processes to improve interpersonal functioning. In contrast, skill-based interventions showed the smallest effect among the five approaches. This suggests that while intrapersonal regulation skills may be taught to support emotional and interpersonal functioning, they may not be sufficiently effective when basic capacities impaired by traumatic experiences are not addressed first. This highlights the importance of multi-phase intervention protocols that can sufficiently address both trauma-processing and skill development in a logical sequence (Ahmadi Forooshani et al., 2022; Chervonsky & Hunt, 2017; English et al., 2012; Izadikhah et al., 2025).

In contrast, effects on interpersonal competence were less robust and showed limited sensitivity to intervention type. Only three types of interventions— relational, skill-based, and movement—were examined for interpersonal competence. Notably, the movement intervention demonstrated the highest effect size among all, although this finding is based on just one study and should be interpreted with caution. This result highlights that expression through movement may uniquely support adolescents’ interpersonal competence. Further research on movement interventions could also strengthen evidence of their effectiveness in enhancing interpersonal competence. Additionally, given the relative paucity of interventions explicitly targeting interpersonal competence, future research should incorporate a wider range of interventions and examine mechanisms specific to competence development, which may yield different outcomes and insights.

The current study found that the mode of intervention significantly moderated the outcome for interpersonal problems but not for interpersonal competence. Interventions that include both individual and group, or conjoint, sessions are more effective at reducing interpersonal problems compared to fully group-based interventions. This aligns with the TF-CBT approach and prior research, indicating that interpersonal difficulties vary across individuals, and that individual sessions allow adolescents to address interpersonal problems with greater personalisation and reduced shame, embarrassment and judgment compared to group interventions (Cohen et al., 2017; Cohen & Mannarino, 2015). Group or conjoint sessions, in turn, provide opportunities for skill practice, validation and relational learning (Pollio & Deblinger, 2018). Hence, these findings underscore the importance of tailoring interventions to adolescent’s specific interpersonal deficits rather than relying on uniform delivery formats, to effectively improve interpersonal functioning among adolescents with posttraumatic stress symptoms.

Finally, the study found that the number of therapeutic sessions does not influence the intervention effect on enhancing interpersonal competence or reducing interpersonal problems. This suggests that increasing the number of sessions cannot enhance the small intervention effects observed in both areas. Notably, the five studies that examined both interpersonal competence and problems produced smaller effect sizes than those observed when each domain was considered separately. This may reflect the diversity of interventions employed (relational, skill-based, and movement-based), each targeting specific aspects of interpersonal functioning, which could diluted aggregated effect sizes across both domains. Neverthessless, this variation provides valuable insight into how different intervention strategies may be more or less effective for addressing interpersonal competence versus interpersonal problems. In particular, it underscores the importance of designing interventions that either focus on a specific domain or deliberately integrate strategies to target both aspects to maximise effectiveness. The lack of studies with follow-up assessments further limits understanding of the long-term effectiveness of current intervention protocols on interpersonal functioning. Therefore, further research should explore alternative interventions to develop comprehensive approaches that simultaneously address both interpersonal difficulties and interpersonal competence for this population.

A key clinical consideration arising from these findings is the sequencing of interventions, specifically whether interpersonal difficulties should be addressed through tailored intervention strategies immediately after addressing stabilisation and trauma-related symptoms. Phase-based models of adult PTSD and CPTSD typically prioritise stabilisation and symptom reduction before engaging in interpersonal work (Ahmadi Forooshani et al., 2022; Cloitre et al., 2013; Izadikhah et al., 2025; Karatzia et al., 2024). As noted above, the present findings suggest that comparable mechanisms and sequencing may benefit adolescent populations. The results indicate that failing to integrate both pathological symptom reduction and skill development approaches may have contributed to the limited efficacy of current interventions for adolescents with trauma histories. The results showed that while the trauma‑focused interventions can indirectly reduce interpersonal problems, they seem to be insufficient for fostering interpersonal competence. This highlights the potential utility of phased or integrated treatment models, in which trauma symptom reduction is followed by, or combined with, targeted interventions addressing interpersonal functioning.

Several limitations should be considered. The relatively small number of eligible studies limits statistical power and generalisability, although this is comparable to other meta-analyses in focusing on PTSD and mood disorders (e.g., Gillies et al., 2012; Lenz & Hollenbaugh, 2015; Phillips et al., 2024; Silverman et al., 2008) and the only other study addressing interpersonal functioning among adolescents with posttraumatic stress symptoms (i.e., Phillips et al., 2024). The lack of follow-up assessments precludes conclusions about the long-term impact of interventions on interpersonal functioning. Additionally, most studies relied on subscales from broader measures rather than instruments specifically designed to assess interpersonal functioning, which may not accurately reflect an individual’s interpersonal functioning. The inability to examine moderators such as gender, age and trauma type, as these factors were not sufficiently explored or highlighted in the included studies, further constrains interpretation.

These limitations point to clear priorities for future search. There is a need for high-quality RCTs that explicitly target interpersonal functioning, employ validated interpersonal measures (e.g., IIP-64,) include follow-up assessments, and examine developmental and demographic moderators. Intervention adapted from adult PTSD and CPTSD research, particular those emphasizing phased treatment and relational skills, may present promising directions for innovation.

Overall, this meta-analysis highlights the need to reassess current interventions for adolescents with posttraumatic stress symptoms. The study demonstrates that current psychological interventions for adolescents with PTSD yield only a small improvement in interpersonal functioning, particularly when such outcomes are not directly targeted, with no strong evidence suggesting long-term benefit. While trauma-focused interventions appear effective in reducing interpersonal problems, they show limited capacity to enhance interpersonal competence. These findings highlight the need for developmentally tailored, empirically tested interventions that explicitly address both dimensions of interpersonal functioning. Advancing this area of research will be critical for improving long-term social and relational outcomes for adolescents with posttraumatic stress symptoms.

## Data Availability

All data analysed in this meta-analysis were obtained from published studies. Extracted datasets and analysis files are available from the corresponding author upon reasonable request.

## Appendix

**Table:**

Database	Date searched	Search Terms	Limits	Records Retrived
PubMed	30/05/2024	((“Adolescent“[MeSH Terms] OR (“adolescen*“[Text Word] OR “teen*“[Text Word] OR “Youth“[Text Word] OR “Young people“[Text Word])) AND ((“stress disorders, post traumatic“[MeSH Terms] OR (“PTSD“[Text Word] OR “CPTSD“[Text Word] OR “trauma*“[Text Word] OR “maltreat*“[Text Word] OR “mistreat*“[Text Word] OR “child abuse“[Text Word] OR “adverse childhood“[Text Word] OR ((“maladaptation“[All Fields] OR “maladaptations“[All Fields] OR “maladaptative“[All Fields] OR “maladapted“[All Fields] OR “maladaption“[All Fields] OR “maladaptive“[All Fields] OR “maladaptively“[All Fields] OR “maladaptiveness“[All Fields]) AND “childhood“[Text Word]) OR “posttraumatic“[Text Word] OR “post-traumatic“[Text Word] OR “post-traumatic“[Text Word])) NOT “Wounds and Injuries“[MeSH Terms]) AND (“Psychotherapy“[MeSH Terms] OR (“intervention*“[Text Word] OR “treat*“[Text Word] OR “therap*“[Text Word] OR “psychotherap*“[Text Word])) AND (“Interpersonal problems“[Text Word] OR “Interpersonal issues“[Text Word] OR “Interpersonal difficulties“[Text Word] OR “Social Adjustment“[Text Word] OR “Social maladjustment“[Text Word] OR “Maladjustment“[Text Word] OR “Relationship Issues“[Text Word] OR “Relationship Problems“[Text Word])) AND ((english[Filter]) AND (2014:2024[pdat])) - Saved search	English, 2014–2024	98
CINAHL	30/05/2024	S1: Adolescen* OR Teen* OR Youth OR “Young people” S2: PTSD OR CPTSD OR Trauma* OR Maltreat* OR Mistreat* OR “Child abuse” OR “Adverse childhood” OR “maladaptive childhood” OR posttraumatic OR “post traumatic” OR “post- traumatic” S3: Intervention* OR Treat* OR Therap* OR Psychotherap* S4: “Interpersonal problems” OR “Interpersonal issues” OR “Interpersonal difficulties” OR “Social Adjustment” OR “Social maladjustment” OR Maladjustment OR “Relationship Issues” OR “Relationship Problems” S1 AND S2 AND S3 AND S4	English, 2014–2024	158
PsycINFO	30/05/2024	S1: Adolescen* OR Teen* OR Youth OR “Young people” S2: PTSD OR CPTSD OR Trauma* OR Maltreat* OR Mistreat* OR “Child abuse” OR “Adverse childhood” OR “maladaptive childhood” OR posttraumatic OR “post traumatic” OR “post- traumatic” S3: Intervention* OR Treat* OR Therap* OR Psychotherap* S4: “Interpersonal problems” OR “Interpersonal issues” OR “Interpersonal difficulties” OR “Social Adjustment” OR “Social maladjustment” OR Maladjustment OR “Relationship Issues” OR “Relationship Problems” S1 AND S2 AND S3 AND S4	English, 2014–2024	202
Scopus	30/05/2025	( TITLE-ABS-KEY ( adolescen* OR teen* OR youth OR “young people” ) ) AND ( TITLE-ABS-KEY ( ptsd OR cptsd OR trauma* OR maltreat* OR mistreat* OR child AND abuse OR adverse AND childhood OR maladaptive AND childhood OR posttraumatic OR “post traumatic” OR post- traumatic ) ) AND ( TITLE-ABS-KEY ( intervention* OR treat* OR therap* OR psychotherap* ) ) AND ( TITLE-ABS-KEY ( “interpersonal problems” OR “interpersonal issues” OR “interpersonal difficulties” OR “social adjustment” OR “social maladjustment” OR maladjustment OR “relationship issues” OR “relationship problems” ) ) AND PUBYEAR > 2013 AND PUBYEAR < 2025 AND ( LIMIT-TO ( LANGUAGE, “english” ) )	English, 2014–2024	71
Google Scholar via *Publish or Perish*	30/05/2025	CPTSD PTSD adolescent “interpersonal problems” intervention	English, 2014–2024	973
PubMed	17/12/2025	(“Adolescent“[MeSH Terms] OR (“adolescen*“[Text Word] OR “teen*“[Text Word] OR “Youth“[Text Word] OR “Young people“[Text Word])) AND ((“stress disorders, post traumatic“[MeSH Terms] OR (“PTSD“[Text Word] OR “CPTSD“[Text Word] OR “trauma*“[Text Word] OR “maltreat*“[Text Word] OR “mistreat*“[Text Word] OR “child abuse“[Text Word] OR “adverse childhood“[Text Word] OR ((“maladaptation“[All Fields] OR “maladaptations“[All Fields] OR “maladaptative“[All Fields] OR “maladapted“[All Fields] OR “maladaption“[All Fields] OR “maladaptive“[All Fields] OR “maladaptively“[All Fields] OR “maladaptiveness“[All Fields]) AND “childhood“[Text Word]) OR “posttraumatic“[Text Word] OR “post-traumatic“[Text Word] OR “post-traumatic“[Text Word])) NOT “Wounds and Injuries“[MeSH Terms]) AND (“Psychotherapy“[MeSH Terms] OR (“intervention*“[Text Word] OR “treat*“[Text Word] OR “therap*“[Text Word] OR “psychotherap*“[Text Word])) AND (“Interpersonal problems“[Text Word] OR “Interpersonal issues“[Text Word] OR “Interpersonal difficulties“[Text Word] OR “Social Adjustment“[Text Word] OR “Social maladjustment“[Text Word] OR “Maladjustment“[Text Word] OR “Relationship Issues“[Text Word] OR “Relationship Problems“[Text Word]) AND (“english“[Language] AND 2024/05/30:2025/12/31[Date - Publication])	English, 2024–2025	21
CINAHL	17/12/2025	S1: Adolescen* OR Teen* OR Youth OR “Young people” S2: PTSD OR CPTSD OR Trauma* OR Maltreat* OR Mistreat* OR “Child abuse” OR “Adverse childhood” OR “maladaptive childhood” OR posttraumatic OR “post traumatic” OR “post- traumatic” S3: Intervention* OR Treat* OR Therap* OR Psychotherap* S4: “Interpersonal problems” OR “Interpersonal issues” OR “Interpersonal difficulties” OR “Social Adjustment” OR “Social maladjustment” OR Maladjustment OR “Relationship Issues” OR “Relationship Problems” S1 AND S2 AND S3 AND S4	English, 2024–2025	39
PsycINFO	17/12/2025	S1: Adolescen* OR Teen* OR Youth OR “Young people” S2: PTSD OR CPTSD OR Trauma* OR Maltreat* OR Mistreat* OR “Child abuse” OR “Adverse childhood” OR “maladaptive childhood” OR posttraumatic OR “post traumatic” OR “post- traumatic” S3: Intervention* OR Treat* OR Therap* OR Psychotherap* S4: “Interpersonal problems” OR “Interpersonal issues” OR “Interpersonal difficulties” OR “Social Adjustment” OR “Social maladjustment” OR Maladjustment OR “Relationship Issues” OR “Relationship Problems” S1 AND S2 AND S3 AND S4	English, 2024–2025	19
Scopus	17/12/2025	( TITLE-ABS-KEY ( adolescen* OR teen* OR youth OR “young people” ) ) AND ( TITLE-ABS-KEY ( ptsd OR cptsd OR trauma* OR maltreat* OR mistreat* OR child AND abuse OR adverse AND childhood OR maladaptive AND childhood OR posttraumatic OR “post traumatic” OR post- traumatic ) ) AND ( TITLE-ABS-KEY ( intervention* OR treat* OR therap* OR psychotherap* ) ) AND ( TITLE-ABS-KEY ( “interpersonal problems” OR “interpersonal issues” OR “interpersonal difficulties” OR “social adjustment” OR “social maladjustment” OR maladjustment OR “relationship issues” OR “relationship problems” ) ) AND PUBYEAR > 2023 AND PUBYEAR < 2026 AND ( LIMIT-TO ( LANGUAGE, “english” ) )	English, 2024–2025	34
Google Scholar via *Publish or Perish*	17/12/2025	CPTSD PTSD adolescent “interpersonal problems” intervention	English, 2024–2025	306
